# Depressive disorders in comorbidity with Multiple Sclerosis. Case study

**DOI:** 10.1192/j.eurpsy.2023.1590

**Published:** 2023-07-19

**Authors:** E. Shaska

**Affiliations:** Acute Care Unit, Psychiatric Hospital “Ali Mihali” Vlore, Vlora, Albania

## Abstract

**Introduction:**

Multiple Sclerosis is a neurodegenerative, demyelinating disease that affects the Central Nervous System. Except for motor dysfunction and sensory deficit, patients suffering from this disorder often have neuropsychiatric symptoms, such as: depressive mood, fatigue, and cognitive impairment. Depression is the most common mental disorder in Multiple Sclerosis, and the risk that MS patients develop depression during their entire life is >50%.

**Objectives:**

Factors impacting on the development of depression

**Methods:**

A regular, clinical study approach has been used on a 49-year old woman, who was diagnosed with depressive Disorder 2 years ago and then Multiple Sclerosis, as well as recent literature on depressive disorders in comorbidity with Multiple Sclerosis.

**Results:**

The factors that considerably impact the development of depression are age, gender, insomnia, cognitive impairment, MS clinical picture, and immunotherapy treatment. Depression was diagnosed at the clinical interview, based on DSM-5 diagnosis criteria and Beck Inventory, whereas MS diagnosis was determined by neurological examination and head MRI. The patient was treated with tricyclic antidepressants, SSRIs, SNRIs, atypical antipsychotics for depression, and teriflunomide for MS. Depression has been recurrent, despite being regularly treated with psychotropic medications

**Conclusions:**

Depressive disorders in comorbidity with multiple sclerosis are often undiagnosed and improperly treated. Many factors influence the development and progression of depression, as well as the Multiple Sclerosis clinical picture, above all. Early diagnosis and optimal treatment of them are essential to control the disease and improve the quality of life.

**Disclosure of Interest:**

None Declared

